# BioWaitIndex: a novel machine learning-derived biomarker for improved prediction of liver transplant waitlist outcomes

**DOI:** 10.3389/frtra.2026.1964045

**Published:** 2026-10-14

**Authors:** Khush Patel, Ashton A. Connor, Mansi Sanghvi, Ahmed Elaileh, Elsie Rizk, Samar Semaan, Youssef E. Dib, Jason Todd, Linda W. Moore, Luma Succar, Caroline J. Simon, Yee Lee Cheah, Constance M. Mobley, Ashish Saharia, Kavish R. Patidar, Sudha Kodali, David W. Victor, Scott Lindberg, Stefano Casarin, Xian C. Li, A. Osama Gaber, R. Mark Ghobrial

**Affiliations:** 1 Department of Surgery, Houston Methodist Hospital, Houston, TX, United States; 2 J. C. Walter Jr. Transplant Center, Houston Methodist Hospital, Houston, TX, United States; 3 Houston Methodist Academic Institute, Houston, TX, United States; 4 Department of Surgery, Weill Cornell Medicine, Cornell University, New York, NY, United States; 5 Department of Medicine, Gastroenterology, Houston Methodist Hospital, Houston, TX, United States; 6 Department of Medicine, Weill Cornell Medicine, Cornell University, New York, NY, United States; 7 Department of Anesthesiology and Critical Care, Houston Methodist Hospital, Houston, TX, United States; 8 Department of Surgery, Immunobiology and Transplant Science Center, Houston Methodist Research Institute, Houston Methodist Hospital, Houston, TX, United States

**Keywords:** clinical validation, liver transplantation, machine learning-derived composite index, MELD, waitlist mortality

## Abstract

**Background:**

Liver transplantation remains limited by organ shortage, necessitating precise waitlist risk stratification. The Model for End-Stage Liver Disease (MELD) remains the cornerstone of liver allocation and effectively predicts near-term mortality when assessed dynamically during waitlist follow-up. However, MELD measured at initial transplant evaluation may not fully capture baseline multisystem vulnerability, particularly among candidates with lower MELD scores who may remain on the waitlist for prolonged periods.

**Methods:**

The BioWaitIndex (BWI) was developed using a random forest model in an NHANES development cohort comprising 13,830 participants. The trained model was first temporally validated in a separate, non-overlapping sample from subsequent NHANES cycles comprising 8,151 participants. It was then independently clinically validated in a separate, non-overlapping, IRB-approved cohort of 1,502 adult liver transplant candidates from Houston Methodist Hospital. In the clinical validation cohort, associations between BWI measured at initial transplant evaluation and subsequent waitlist outcomes were assessed using competing-risk regression.

**Results:**

In multivariable competing-risks analyses with BWI and MELD both measured at initial evaluation, each unit increase in BWI was associated with a greater likelihood of transplantation (sHR 1.02, 95% CI 1.01–1.03; *p* < 0.001) and a lower risk of waitlist death or clinical deterioration (sHR 0.97, 95% CI 0.97–0.98; *p* < 0.001). Adding BWI to MELD at evaluation improved net reclassification for waitlist mortality by 31.2% (*p* < 0.001) and for transplantation by 11.1% (*p* = 0.026), with consistent associations across sexes.

**Conclusion:**

BWI may complement dynamic MELD assessment by providing earlier information about baseline multisystem vulnerability and identifying candidates whose risk may not be fully reflected by their MELD score at evaluation.

## Introduction

Liver transplantation (LT) remains the definitive therapeutic intervention for end-stage liver disease (ESLD). Despite record growth in transplant volume, a persistent gap between organ supply and demand continues to challenge the transplant community: in 2023 alone, nearly 14,700 candidates were added to the liver transplant waiting list in the United States, while approximately 10,660 transplants were performed, underscoring the critical need for precise and equitable allocation strategies (1). These strategies must prioritize both fairness and clinical utility to ensure that patients at the highest risk of waitlist mortality receive timely transplants.

Current waitlist prioritization relies primarily on the Model for End-Stage Liver Disease (MELD) score and its subsequent refinements (MELD-Na, MELD 3.0, and GEMA), which have anchored LT allocation since 2002. MELD significantly improved the prediction of 90-day waitlist mortality through objective measures of liver and kidney dysfunction (bilirubin, INR, creatinine, and sodium) (2–7). By design, MELD prioritizes acute severity from laboratory values obtained near the time of organ offer, and was not intended to capture a patient's longer-term trajectory or underlying physiologic reserve (8–10).

Prior efforts to quantify systemic reserve beyond organ-specific scores have relied on frailty indices such as the Liver Frailty Index and Fried Frailty Phenotype (10–13). While these tools have demonstrated prognostic value, they require in-person physical performance assessments (grip strength, chair stands, balance testing) that may not be feasible in severely deconditioned patients, are subject to interobserver variability, and are not routinely captured in the electronic medical record (12, 14–16). Alternatively, molecular assays such as DNA methylation-based measures offer objective quantification of multisystem physiologic status but are constrained by small sample sizes, specialized processing requirements, high cost, and lack of scalability to routine clinical workflows (17–19).

Biomarker-based measures of multisystem physiologic status derived from the National Health and Nutrition Examination Survey (NHANES), a nationally representative survey of the general, community-dwelling US population, and subsequently applied to temporally distinct or independent clinical cohorts, represent an established translational framework. NHANES-derived indices have demonstrated associations with mortality, disease burden, and prognosis across diverse populations, including patients with diabetes, cardiovascular disease, obesity, nonalcoholic fatty liver disease, inflammatory disorders, cancer, and neurologic disease (20–33). Building on this established framework, we developed the BioWaitIndex (BWI), a machine learning-derived composite index designed to integrate routinely collected clinical data at the time of LT evaluation. BWI incorporates twelve biomarkers available in the electronic medical record spanning pulmonary, cardiovascular, kidney, metabolic, inflammatory, nutritional, and hematologic domains to characterize a candidate's systemic physiologic status. The model was developed in an NHANES cohort of 13,830 participants, temporally validated in a separate, non-overlapping sample from subsequent NHANES cycles comprising 8,151 participants, and subsequently independently clinically validated in a separate, non-overlapping institutional cohort of 1,502 adult liver transplant candidates. Its application to liver transplant waitlist risk stratification has not previously been evaluated. We therefore examined whether BWI could provide clinically useful prognostic information at initial transplant evaluation and complement MELD-based risk assessment.

## Methods

### Development, temporal-validation, and independent clinical-validation cohorts

#### Development cohort

Data for model development were obtained from the National Health and Nutrition Examination Survey (NHANES), a nationally representative cross-sectional survey of the noninstitutionalized U.S. civilian population conducted by the National Center for Health Statistics. The development cohort included participants aged 20–90 years (median 43, IQR 31-63) with complete data for the selected biomarkers (*n* = 13,830) designated “NHANES III” within the BioAge package. Data extraction and harmonization were performed using the BioAge R package (21). For this analysis, NHANES data were treated as a simple random sample rather than analyzed using the survey's complex sampling design.

### Temporal-validation cohort

Temporal evaluation was performed in a separate sample from subsequent continuous NHANES cycles (*n* = 8,151), corresponding to the dataset designated “NHANES IV” within the BioAge package.

### Independent clinical-validation cohort

The clinical application cohort comprised 1,502 adult candidates listed for primary liver transplantation at the Houston Methodist J.C. Walter Jr. Transplant Center between June 2016 and November 2022, with outcomes followed through December 2024. Time zero for the waitlist analyses was the date of formal listing. The institutional cohort study was approved by the Houston Methodist Institutional Review Board under protocol Pro00000587; it was not registered in a public registry, and no separate analysis protocol was published. Patients and the public were not involved in the design, conduct, or reporting of the study. The overall study design, including model development, temporal evaluation, and application to the liver transplant waitlist cohort within a competing-risks framework, is summarized in Figure 1. No formal sample-size calculation was performed; all eligible candidates were included.

**Figure 1:**
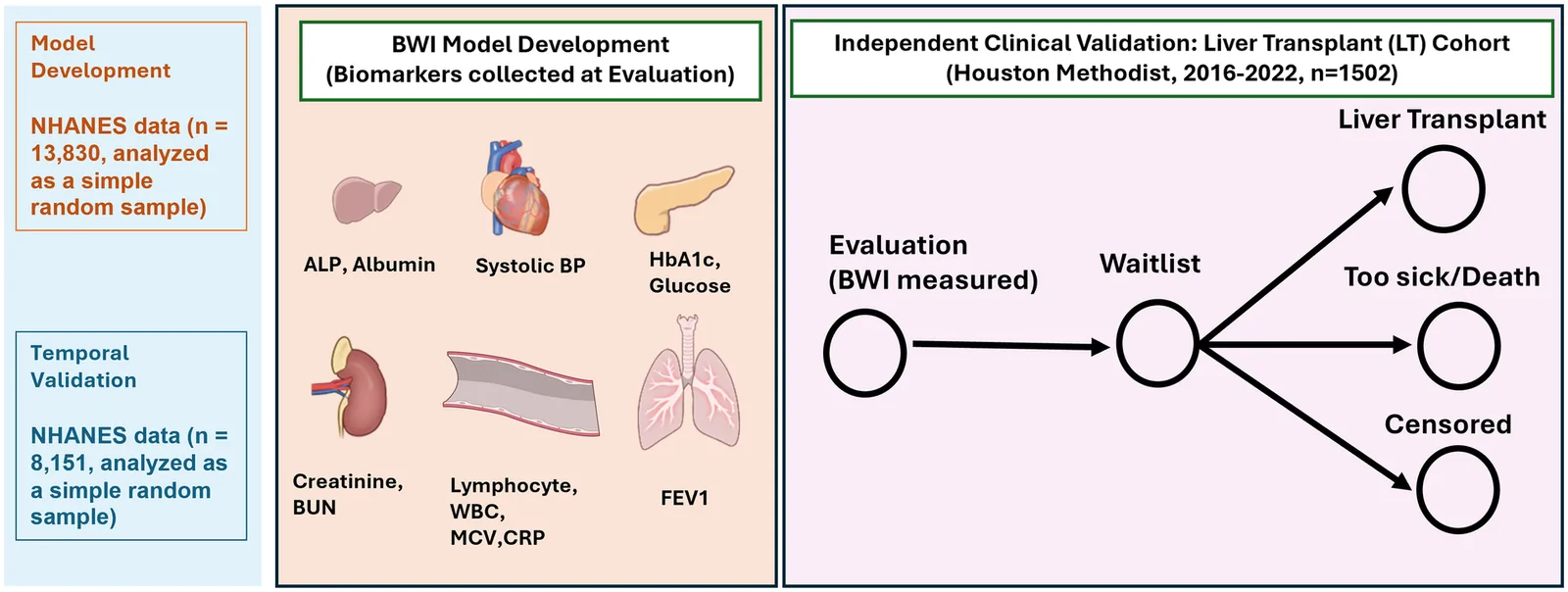
Study design and BioWaitIndex development framework. A random forest regression model was developed in the National Health and Nutrition Examination Survey (NHANES) development cohort (*n* = 13,830) to predict chronological age using 12 routinely collected biomarkers spanning pulmonary, cardiovascular, renal, metabolic, inflammatory, nutritional, and hematologic domains. BioWaitIndex (BWI) was calculated as the difference between model-predicted age and chronological age, yielding a continuous measure of multisystem physiologic status. The trained model was then temporally validated in a separate, non-overlapping sample from subsequent NHANES cycles (*n* = 8,151). Independent clinical validation was subsequently performed in a separate institutional cohort of 1,502 adult liver transplant candidates listed at Houston Methodist between 2016 and 2022. In the clinical-validation cohort, BWI was calculated using biomarkers collected during the initial transplant evaluation. Transplantation and death or removal because of clinical deterioration were analyzed as competing waitlist outcomes. Candidates who remained on the waitlist, transferred care, or were removed for reasons other than death or clinical deterioration were censored at their last follow-up.

### Outcome definitions

Waitlist outcomes were harmonized from administrative removal codes into three categories for survival analysis. Transplant (event of interest): living and deceased donor transplants, including transplants performed at other centers and patients who died during the transplant procedure. Death or deterioration (competing risk): patients who died while waiting or were removed due to clinical deterioration. Censored: patients transferred to another center (censored at time of transfer) or removed for non-mortality administrative reasons including condition improvement, refusal of transplant, inability to contact, or medical unsuitability without immediate mortality (censored at time of removal).

### Biomarker selection and BWI development

Twelve biomarkers routinely measured in clinical practice and spanning multiple physiologic domains (nutritional, inflammatory, metabolic, renal, cardiovascular, pulmonary, and hematologic) were selected for constructing the BioWaitIndex (BWI): albumin, C-reactive protein (natural log-transformed), white blood cell count, glucose, glycated hemoglobin (HbA1c), alkaline phosphatase, blood urea nitrogen, creatinine, systolic blood pressure, forced expiratory volume in 1 s (FEV1), lymphocyte count, and mean corpuscular volume. A random forest regression model (500 trees, minimum node size of 5; 4 variables considered at each split using randomForest package in R) was trained on the NHANES III dataset using chronological age as the training target to anchor the model's learned representation of multisystem physiologic status. BWI was calculated as the difference between the model's predicted value and chronological age. Positive values indicate a biomarker profile predicted to be older than expected for chronological age, whereas negative values indicate a profile predicted to be younger than expected. Importantly, BWI is interpreted not as an age estimate but as a composite measure of multisystem physiologic status. Feature importance was assessed in the liver transplant cohort using permutation-based variable importance: each biomarker was randomly permuted and the resulting increase in mean squared error for predicting chronological age at evaluation was quantified, identifying which biomarkers most influenced BWI in the population of interest. All model development, variable selection, and hyperparameter specification were performed using the NHANES III development cohort. The fitted model was subsequently applied without retraining to the temporal-validation and independent clinical-validation cohorts.

### Statistical analysis

Baseline characteristics are summarized as medians with interquartile ranges for continuous variables and frequencies with percentages for categorical variables. Comparisons across the three waitlist outcome groups were performed using Kruskal–Wallis tests for continuous variables and Fisher's exact tests for categorical variables. Missing biomarker values in the institutional cohort were imputed using multiple imputation by chained equations (mice package in R) with predictive mean matching for continuous variables, five iterations, and 50 imputed datasets (random seed 42); the imputation model included all variables available at the evaluation timepoint except chronological age, and the first completed dataset was used for the primary analyses. The extent of missingness for each variable and imputation diagnostics, comparing observed and completed distributions by means, standard deviations, and Kolmogorov–Smirnov tests, are provided in Supplementary Table S2. Because the missing-at-random assumption cannot be verified, sensitivity analyses were performed in complete cases and in candidates with observed versus imputed FEV1. All analyses were conducted using R version 4.2.1. Statistical tests were two-sided, with *p* < 0.05 considered statistically significant. To characterize mortality patterns across BWI strata, candidates who died or were removed because of clinical deterioration were stratified by median BWI. MELD scores at the time of the outcome and waitlist durations were compared between the two groups using Wilcoxon rank-sum tests.

### Multivariable competing-risk analysis

The association between BWI at the time of evaluation and the two primary waitlist outcomes (transplantation and death/deterioration) was evaluated using competing risks regression (Fine-Gray subdistribution hazard model; cmprsk package). In the primary analysis, BWI and MELD both measured at initial transplant evaluation were entered jointly into a multivariable model adjusted for candidate sex and race/ethnicity (White Non-Hispanic, Black Non-Hispanic, Hispanic, Asian Non-Hispanic, and Other/Unknown) (Table 2). A secondary analysis paired BWI at evaluation with MELD assessed at the time of the outcome; because this model incorporates information measured after baseline, it is presented as a descriptive association rather than a baseline prediction model (Table 3). To benchmark BWI against established physiologic indices, separate competing-risk models were fitted for each metric, each individually adjusted for sex and race/ethnicity; the biomarker composition of each comparator index, including adaptations due to data availability, is detailed in the Supplementary Material. Subdistribution hazard ratios with 95% confidence intervals are reported for all models. Cumulative incidence functions were constructed to visualize the probability of each outcome over time across BWI strata, accounting for competing events. Because predicted-minus-chronological age metrics may show a systematic dependence on chronological age arising from regression to the mean, both models were repeated with chronological age at evaluation added as a covariate. As a complement to the subdistribution hazard models, cause-specific Cox models were fitted for each outcome with the competing event treated as censoring, adjusted for sex and race/ethnicity.

**Table 1:** Baseline characteristics of liver transplant candidates by waitlist outcome.

Variable	Overall, *N* = 1,502	Censored/Other, *N* = 294	Death or Clinical Deterioration, *N* = 431	Transplantation, *N* = 777	p-value
Age at Listing, years; median (IQR)	59.0 (50.0, 66.0)	58.0 (50.0, 65.0)	63.0 (55.0, 68.0)	58.0 (48.0, 65.0)	**<0** **.** **001**
Sex, n (%)					0.976
Male	900 (60%)	175 (60%)	260 (60%)	465 (60%)	
Female	602 (40%)	119 (40%)	171 (40%)	312 (40%)	
Race and Ethnicity, n (%)					0.182
Non-Hispanic White	951 (63%)	196 (67%)	275 (64%)	480 (62%)	
Hispanic	348 (23%)	66 (22%)	105 (24%)	177 (23%)	
Non-Hispanic Black	118 (7.9%)	14 (4.8%)	30 (7.0%)	74 (9.5%)	
Non-Hispanic Asian	64 (4.3%)	15 (5.1%)	13 (3.0%)	36 (4.6%)	
Other/Unknown	21 (1.4%)	3 (1.0%)	8 (1.9%)	10 (1.3%)	
Primary Diagnosis, n (%)					**<0**.**001**
Alcohol-Associated Liver Disease	486 (32%)	115 (39%)	101 (23%)	270 (35%)	
MASH/Fatty Liver	298 (20%)	42 (14%)	107 (25%)	149 (19%)	
HCC/Malignancy	268 (18%)	48 (16%)	98 (23%)	122 (16%)	
Other/Unknown	162 (11%)	36 (12%)	42 (9.7%)	84 (11%)	
Hepatitis C	143 (9.5%)	25 (8.5%)	48 (11%)	70 (9.0%)	
Autoimmune/Cholestatic	118 (7.9%)	24 (8.2%)	30 (7.0%)	64 (8.2%)	
Hepatitis B	27 (1.8%)	4 (1.4%)	5 (1.2%)	18 (2.3%)	
MELD at Evaluation, median (IQR)	19.0 (11.0, 29.0)	14.0 (9.0, 20.3)	18.0 (10.8, 27.0)	22.0 (13.0, 32.0)	**<0**.**001**
Waitlist Duration, days; median (IQR)	232 (27, 616)	1,010 (397, 1,470)	202 (51, 545)	105 (12, 391)	**<0**.**001**
BioWaitIndex (BWI) at evaluation; median (IQR)	2.8 (−5.1, 11.3)	2.8 (−5.4, 9.6)	0.4 (−7.0, 7.1)	5.0 (−3.9, 14.9)	**<0**.**001**

Values are presented as median (interquartile range) for continuous variables and n (%) for categorical variables. Comparisons across outcome groups were performed using the Kruskal–Wallis rank-sum test for continuous variables and Fisher's exact test for categorical variables. The censored/other group included candidates who remained on the waitlist, transferred care, or were removed for reasons other than death or clinical deterioration.

BWI, BioWaitIndex; HCC, hepatocellular carcinoma; IQR, interquartile range; MASH, metabolic dysfunction-associated steatohepatitis; MELD, model for end-stage liver disease.

Bold values indicate statistical significance (*p* < 0.05).

**Table 2:** Multivariable Fine-Gray competing-risk models of waitlist outcomes with BWI and MELD measured at initial transplant evaluation.

Variable	Coefficient	SE	sHR	95% CI	p-value
Panel A: Outcome: Transplantation					
MELD at evaluation	0.043	0.005	1.04	1.03, 1.05	**<0** **.** **001**
BWI at evaluation	0.018	0.004	1.02	1.01, 1.03	**<0**.**001**
Sex (Male)	0.103	0.087	1.11	0.93, 1.31	0.24
Race: Non-Hispanic Black	0.045	0.161	1.05	0.76, 1.43	0.78
Race: Hispanic	−0.063	0.104	0.94	0.77, 1.15	0.54
Race: Non-Hispanic Asian	0.112	0.188	1.12	0.77, 1.62	0.55
Race: Other/Unknown	−0.410	0.422	0.66	0.29, 1.52	0.33
Panel B: Outcome: Death or Clinical Deterioration					
MELD at evaluation	0.010	0.006	1.01	1.00, 1.02	0.095
BWI at evaluation	−0.027	0.004	0.97	0.97, 0.98	**<0**.**001**
Sex (Male)	−0.076	0.112	0.93	0.74, 1.15	0.49
Race: Non-Hispanic Black	−0.086	0.230	0.92	0.58, 1.44	0.71
Race: Hispanic	0.103	0.132	1.11	0.86, 1.44	0.43
Race: Non-Hispanic Asian	−0.249	0.291	0.78	0.44, 1.38	0.39
Race: Other/Unknown	0.411	0.370	1.51	0.73, 3.11	0.27

BWI, BioWaitIndex; CI, confidence interval; MELD, Model for End-Stage Liver Disease; SE, standard error; sHR, subdistribution hazard ratio.

Estimates were obtained from multivariable Fine–Gray competing-risk models jointly including BWI and MELD measured at initial transplant evaluation, with adjustment for sex and race and ethnicity. For each model, the alternative waitlist outcome was treated as the competing event. Models include the 1,259 candidates with a MELD score recorded at initial evaluation (687 transplantations; 344 deaths or clinical deteriorations). Reference categories were female sex and Non-Hispanic White race and ethnicity. Age-adjusted estimates are provided in Supplementary Table S9.

Bold values indicate statistical significance (*p* < 0.05).

**Table 3:** Secondary analysis: multivariable Fine-Gray competing-risk models with BWI at evaluation and MELD at the time of outcome.

Variable	Coefficient	SE	sHR	95% CI	p-value
Panel A: Outcome: Transplantation					
MELD at time of outcome	0.028	0.003	1.03	1.02, 1.03	**<0** **.** **001**
BWI at evaluation	0.025	0.003	1.03	1.02, 1.03	**<0**.**001**
Sex (Male)	0.161	0.081	1.17	1.00, 1.38	0.046
Race: Non-Hispanic Black	0.125	0.140	1.13	0.86, 1.49	0.37
Race: Hispanic	−0.098	0.094	0.91	0.75, 1.09	0.30
Race: Non-Hispanic Asian	0.115	0.168	1.12	0.81, 1.56	0.50
Race: Other/Unknown	−0.270	0.404	0.76	0.35, 1.68	0.50
Panel B: Outcome: Death or Clinical Deterioration					
MELD at time of outcome	0.025	0.005	1.03	1.02, 1.03	**<0**.**001**
BWI at evaluation	−0.028	0.004	0.97	0.96, 0.98	**<0**.**001**
Sex (Male)	−0.025	0.101	0.98	0.80, 1.19	0.81
Race: Non-Hispanic Black	−0.032	0.199	0.97	0.66, 1.43	0.87
Race: Hispanic	0.112	0.117	1.12	0.89, 1.41	0.34
Race: Non-Hispanic Asian	−0.323	0.265	0.72	0.43, 1.22	0.22
Race: Other/Unknown	0.386	0.379	1.47	0.70, 3.09	0.31

BWI, BioWaitIndex; CI, confidence interval; MELD, Model for End-Stage Liver Disease; SE, standard error; sHR, subdistribution hazard ratio.

Estimates were obtained from multivariable Fine–Gray competing-risk models jointly including BWI measured at initial transplant evaluation and MELD assessed at the time of the outcome, with adjustment for sex and race and ethnicity. Because MELD at the time of the outcome is measured after baseline, this analysis is presented as a descriptive association rather than a baseline prediction model. For each model, the alternative waitlist outcome was treated as the competing event. Models include the 1,480 candidates with a MELD score recorded at the time of the outcome; 22 transplanted candidates without a recorded MELD at transplantation were excluded (755 transplantations; 431 deaths or clinical deteriorations). Reference categories were female sex and Non-Hispanic White race and ethnicity. Age-adjusted estimates are provided in Supplementary Table S10.

Bold values indicate statistical significance (*p* < 0.05).

### Evaluation of incremental predictive value

To assess the added prognostic value of the BioWaitIndex (BWI) beyond the standard Model for End-Stage Liver Disease (MELD) score, we utilized a competing risks framework to fit Fine-Gray subdistribution hazard models for two distinct outcomes: liver transplantation and death or removal due to clinical deterioration. We compared a reference model incorporating MELD at initial evaluation, adjusted for candidate sex and race/ethnicity, against a model that additionally included BWI at evaluation. The improvement in risk stratification at one year was quantified using continuous net reclassification improvement (NRI) and integrated discrimination improvement (IDI) metrics. The NRI quantified the net direction of changes in predicted risk among candidates who experienced and did not experience the outcome, whereas the IDI measured the improvement in separation of average predicted risks between these groups. To ensure robust estimation, 95% confidence intervals and p-values for both metrics were derived using bootstrap resampling with 1,000 iterations (riskRegression and survival packages in R). A secondary comparison using MELD at the time of the outcome as the reference was also performed. Calibration of the BWI-based models at one year was assessed by comparing predicted risk with the observed Aalen–Johansen cumulative incidence across deciles of predicted risk.

## Results

### Model development, temporal validation, and independent clinical validation

BWI was developed using a random forest regression model trained exclusively in the NHANES III development cohort of 13,830 participants. Temporal validation was then performed in a separate, non-overlapping sample of 8,151 participants from subsequent NHANES cycles. In this temporal-validation cohort, BWI was significantly associated with all-cause mortality (HR 1.28, 95% CI 1.08–1.51; *p* = 0.004). The proportion of participants with BWI beyond ±2 SD of the mean was 4.6% in NHANES III and 4.2% in NHANES IV and ranged from 2.7% to 5.0% across age decades, consistent with a normal distribution and without evidence of excess extreme values (Supplementary Table S1).

The model was then applied to an independent institutional cohort of 1,502 adult liver transplant candidates (Table 1). The waitlist cohort had a median age of 59.0 years (IQR 50.0-66.0), 60% were male, and 63% were Non-Hispanic White, followed by Hispanic (23%) and Non-Hispanic Black (7.9%). The median MELD score at evaluation was 19.0 (IQR 11.0−29.0). The primary indications for LT waitlisting were alcohol-associated liver disease (32%), MASH/fatty liver disease (20%), and HCC/malignancy (18%). At the end of follow-up, 777 candidates (52%) underwent transplantation, 431 (29%) died or were removed because of clinical deterioration, and 294 (20%) were censored. The median waitlist duration was 231.5 days (IQR 27.0-615.8). FEV1 was more frequently missing; imputed distributions closely matched observed distributions for all biomarkers except CRP, where the completed distribution was modestly lower (Supplementary Table S2). In sensitivity analyses, the association between BWI and death or clinical deterioration was consistent across the full imputed cohort, complete cases, and candidates with observed or imputed FEV1 (sHR 0.97–0.98, all *p* ≤ 0.005), and the association with transplantation was of the same direction and magnitude in all subsets (Supplementary Table S3). BWI was intentionally trained in a broad general-population cohort rather than in patients with end-stage liver disease. This design allowed the model to establish expected age-related patterns across multiple biomarker systems without learning liver-disease-specific abnormalities; BWI therefore reflects the degree to which an individual candidate's biomarker profile deviates from these reference patterns. The intended physiologic contrast between the development and application cohorts was evident in Supplementary Table S4. Compared with the NHANES development cohort, LT candidates had lower albumin concentrations (median 3.0 vs. 4.1 g/dL), higher alkaline phosphatase levels (132.5 vs. 81.0 U/L), higher C-reactive protein concentrations (1.3 vs. 0.2 mg/dL), and higher serum creatinine levels (1.0 vs. 0.8 mg/dL; all *p* < 0.001).

### Relationship with MELD and biomarker feature importance

BWI and MELD at the time of event showed a modest but statistically significant positive correlation (r = 0.26, *p* < 0.001; Figure 2a), with low shared variance (R^2^ ≈ 0.07), indicating that the two metrics capture related but largely distinct dimensions of candidate status. Permutation-based feature importance computed in the liver transplant cohort identified the key physiologic drivers of BWI (Figure 2b). Forced expiratory volume in 1 s (FEV1), systolic blood pressure, and blood urea nitrogen emerged as the leading contributors, indicating that BWI captures systemic physiologic status across pulmonary, cardiovascular, and renal domains rather than relying on liver-specific metrics. The distribution of individual BWI component biomarkers across waitlist outcome groups is presented in Supplementary Table S5.

**Figure 2:**
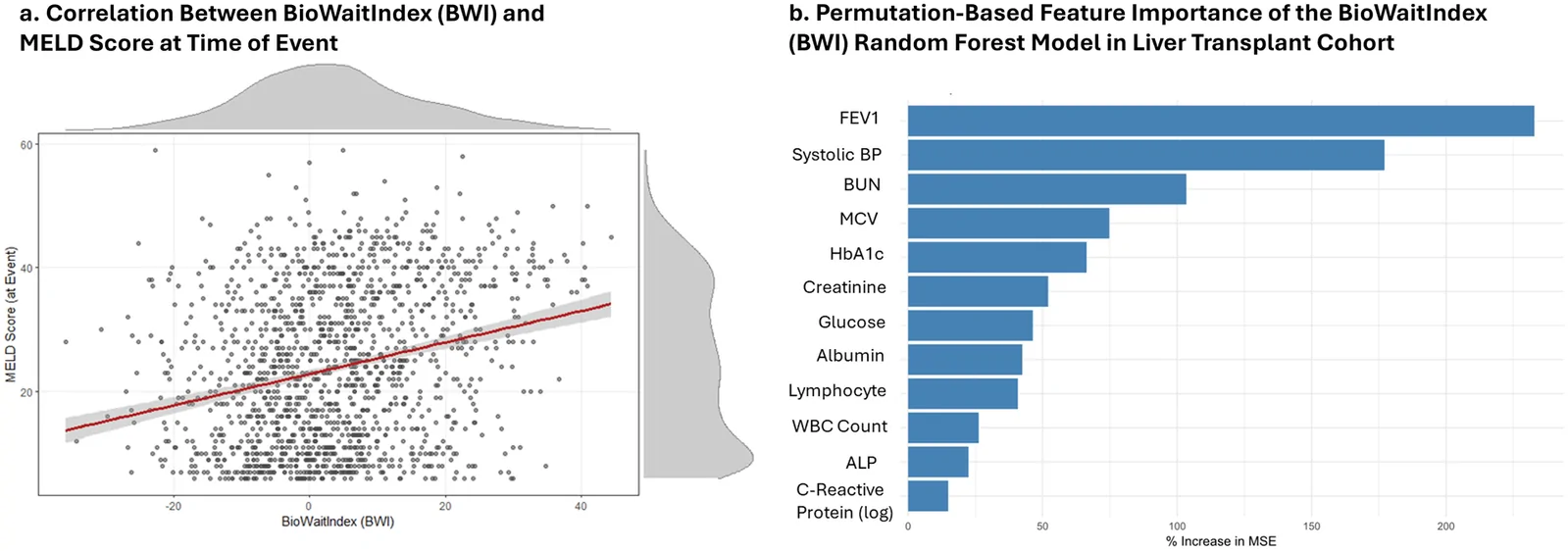
Relationship of BioWaitIndex With MELD and model feature importance. **(a)** Scatterplot with marginal density distributions showing the relationship between BWI measured at initial evaluation and Model for End-Stage Liver Disease (MELD) score assessed at the time of the outcome. A modest positive correlation was observed (r = 0.26; *p* < 0.001), with limited shared variance (r^2^ = 0.07). The fitted regression line is shown with its 95% confidence band. The dispersion of values indicates that candidates with similar MELD scores may have different BWI values, consistent with the two measures capturing related but distinct aspects of candidate status. **(b)** Permutation-based feature importance in the BWI random forest model, quantified as the percentage increase in mean squared error after random permutation of each variable. Importance was computed in the liver transplant cohort by permuting each biomarker and measuring the increase in mean squared error for predicting chronological age at evaluation; higher values indicate a greater influence on BWI in this population. Forced expiratory volume in 1 s, systolic blood pressure, and blood urea nitrogen had the highest permutation importance.

### Multivariable analysis of waitlist outcomes

In the primary multivariable Fine-Gray model, with BWI and MELD both measured at initial evaluation and adjusted for sex and race and ethnicity, higher BWI was associated with a greater subdistribution hazard of transplantation (sHR 1.02, 95% CI 1.01–1.03; *p* < 0.001) and a lower subdistribution hazard of death or clinical deterioration (sHR 0.97, 95% CI 0.97–0.98; *p* < 0.001) (Table 2). MELD at evaluation was associated with transplantation (sHR 1.04, 95% CI 1.03–1.05; *p* < 0.001) but not with death or clinical deterioration (sHR 1.01, 95% CI 1.00–1.02; *p* = 0.095). BWI remained associated with both outcomes within low (< 20) and high (≥ 20) MELD strata (Supplementary Table S6).

Separate competing-risk models showed the same directional pattern: BWI was associated with the two competing outcomes in opposite directions, whereas MELD assessed at the time of the outcome was positively associated with both outcomes; MELD at initial evaluation was associated with transplantation but not with subsequent death or clinical deterioration (Supplementary Tables S7,8). This difference by assessment timing is consistent with MELD's established role as a dynamic predictor of short-term, approximately 90-day mortality; a single MELD value obtained at initial evaluation may not capture changes in clinical acuity later in the waitlist course.

In the secondary analysis pairing BWI at evaluation with MELD at the time of the outcome, both measures were independently associated with waitlist outcomes (Table 3): BWI was associated with a greater subdistribution hazard of transplantation (sHR 1.03, 95% CI 1.02–1.03; *p* < 0.001) and a lower subdistribution hazard of death or clinical deterioration (sHR 0.97, 95% CI 0.96-0.98; *p* < 0.001), whereas MELD at the time of the outcome was positively associated with both. Because transplantation removes a candidate from subsequent risk of waitlist death and is influenced by allocation priority, clinical severity, organ availability, and center-level decision-making, a lower subdistribution hazard alone does not establish a lower rate of death among candidates remaining at risk. In complementary cause-specific models, higher BWI remained associated with a lower hazard of death or clinical deterioration among candidates still at risk (Supplementary Table S11).

After additional adjustment for chronological age at evaluation, BWI remained independently associated with both outcomes, with attenuated estimates (sHR 1.01, 95% CI 1.00–1.02, *p* = 0.004 for transplantation; sHR 0.99, 95% CI 0.98–1.00, *p* = 0.028 for death or clinical deterioration, with MELD at evaluation; Supplementary Tables S9,10).

### Incremental predictive value and reclassification

To assess whether BWI provided incremental prognostic information beyond MELD when both were measured at initial evaluation, we evaluated continuous NRI and IDI at one year. For death or clinical deterioration, adding BWI to the MELD-based model improved risk reclassification (NRI 0.312, 95% CI 0.161–0.460; *p* < 0.001) and discrimination (IDI 0.018; *p* < 0.001). For transplantation, adding BWI improved reclassification (NRI 0.111; *p* = 0.026) and discrimination (IDI 0.012; *p* < 0.001). A secondary analysis using MELD at the time of the outcome as the reference gave similar results (NRI 0.309 and 0.231 respectively). Calibration plots for the BWI-based models at one year are shown in Supplementary Figure S1.

### Quartile and cumulative incidence analysis

Analysis of outcome proportions by BWI quartiles showed monotonic separation at the time of evaluation (Figure 3b): transplant likelihood increased progressively from Q1 (57%) to Q4 (80%), while waitlist mortality decreased correspondingly from Q1 (43%) to Q4 (20%). These descriptive proportions reflect both the occurrence of the outcome and removal from risk through the competing event of transplantation and therefore should not be interpreted as demonstrating a protective effect of higher BWI. In contrast, stratification by MELD score yielded non-monotonic, inconsistent separation across quartiles (Figure 3a). Candidates with above-median BWI had a higher cumulative incidence of transplantation and a lower cumulative incidence of death or clinical deterioration than those with below-median BWI, with separation apparent early after waitlist entry (Figure 4a). MELD measured at initial evaluation produced less distinct separation of the cumulative incidence curves (Figure 4b), whereas MELD assessed at the time of the outcome separated transplantation but did not show the opposing outcome pattern observed with BWI (Figure 4c).

**Figure 3:**
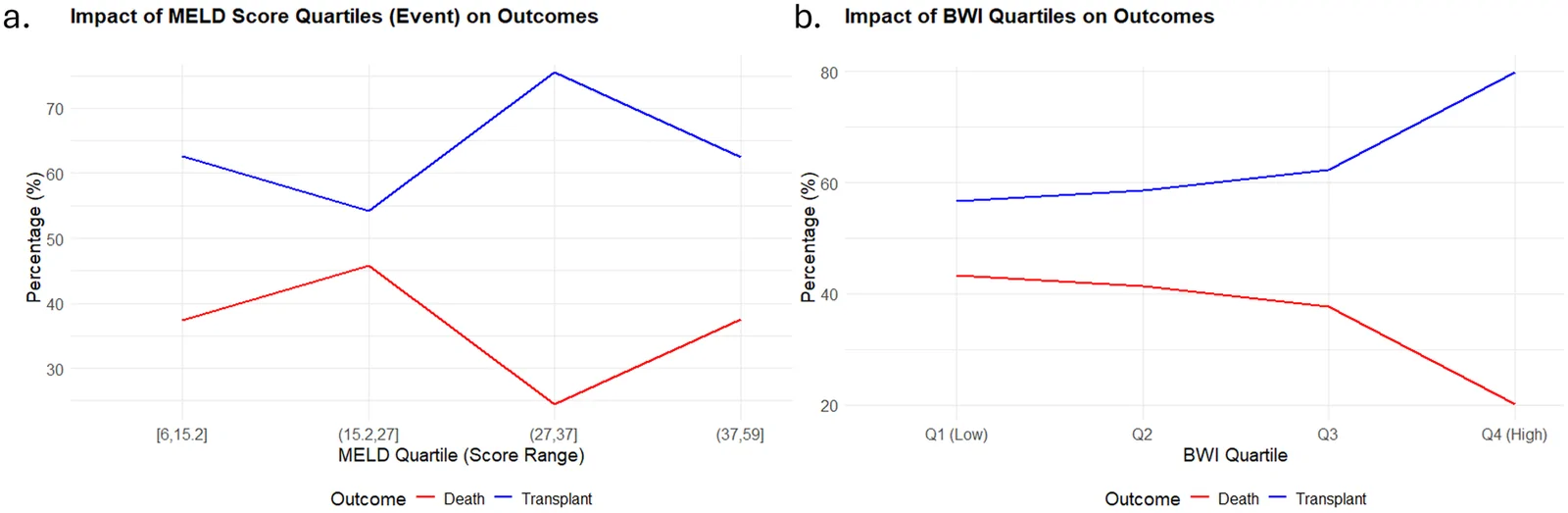
Observed waitlist outcomes by MELD-at-outcome and BWI-at-evaluation quartiles. Percentages of candidates who underwent transplantation or experienced death or removal because of clinical deterioration within each score quartile. **(a)** MELD assessed at the time of the outcome showed nonmonotonic variation in the proportions of transplantation and death or clinical deterioration across quartiles. **(b)** BWI measured at initial evaluation demonstrated a graded pattern: the proportion undergoing transplantation increased from 57% in quartile 1 to 80% in quartile 4, whereas death or clinical deterioration decreased from 43% to 20%.

**Figure 4:**
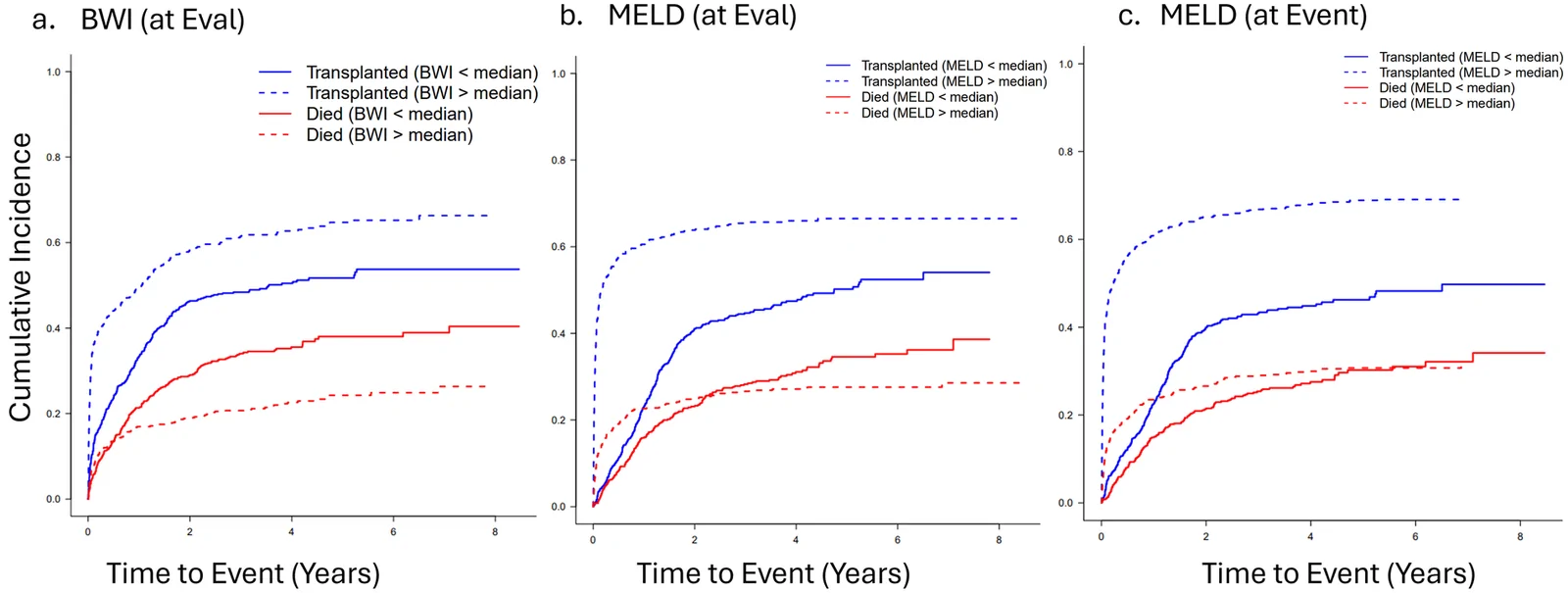
Cumulative incidence of waitlist outcomes stratified by BioWaitIndex and MELD. Cumulative incidence functions for transplantation and death or removal because of clinical deterioration, with the alternative outcome treated as a competing event. Candidates were stratified above and below the median value of each measure. **(a)** Candidates with above-median BWI at initial evaluation had a higher cumulative incidence of transplantation and a lower cumulative incidence of death or clinical deterioration than candidates with below-median BWI. **(b)** MELD measured at initial evaluation separated the transplantation curves but not the death or clinical deterioration curves, which crossed over follow-up. In the primary multivariable model (Table 2), MELD at evaluation was not significantly associated with death or clinical deterioration (sHR 1.01; *p* = 0.095). **(c)** Higher MELD assessed at the time of the outcome was associated with separation of the transplantation curves.

### Characterization of waitlist mortality by BWI group

To further characterize the divergent mortality patterns, we examined MELD scores and waitlist duration among the 431 patients who died or were removed for clinical deterioration, stratified by BWI median split. Low BWI patients accounted for a disproportionate share of waitlist deaths (256/431, 59.4%) despite comprising half the cohort, with a mortality rate of 34.6% compared to 23.6% among high BWI patients. Low BWI patients who died had significantly lower MELD scores at the time of death (median 22 vs. 30, *p* < 0.001) (Figure 5). Despite these lower MELD scores, low BWI patients who died spent significantly longer on the waitlist (median 269 vs. 121 days, *p* = 0.001). A dose-response relationship was observed across BWI quartiles: among deaths, median MELD at event increased progressively from Q1 (19) to Q4 (34), while median time on the waitlist decreased from 286 to 88 days (Kruskal–Wallis *p* < 0.001 and *p* = 0.003, respectively).

**Figure 5:**
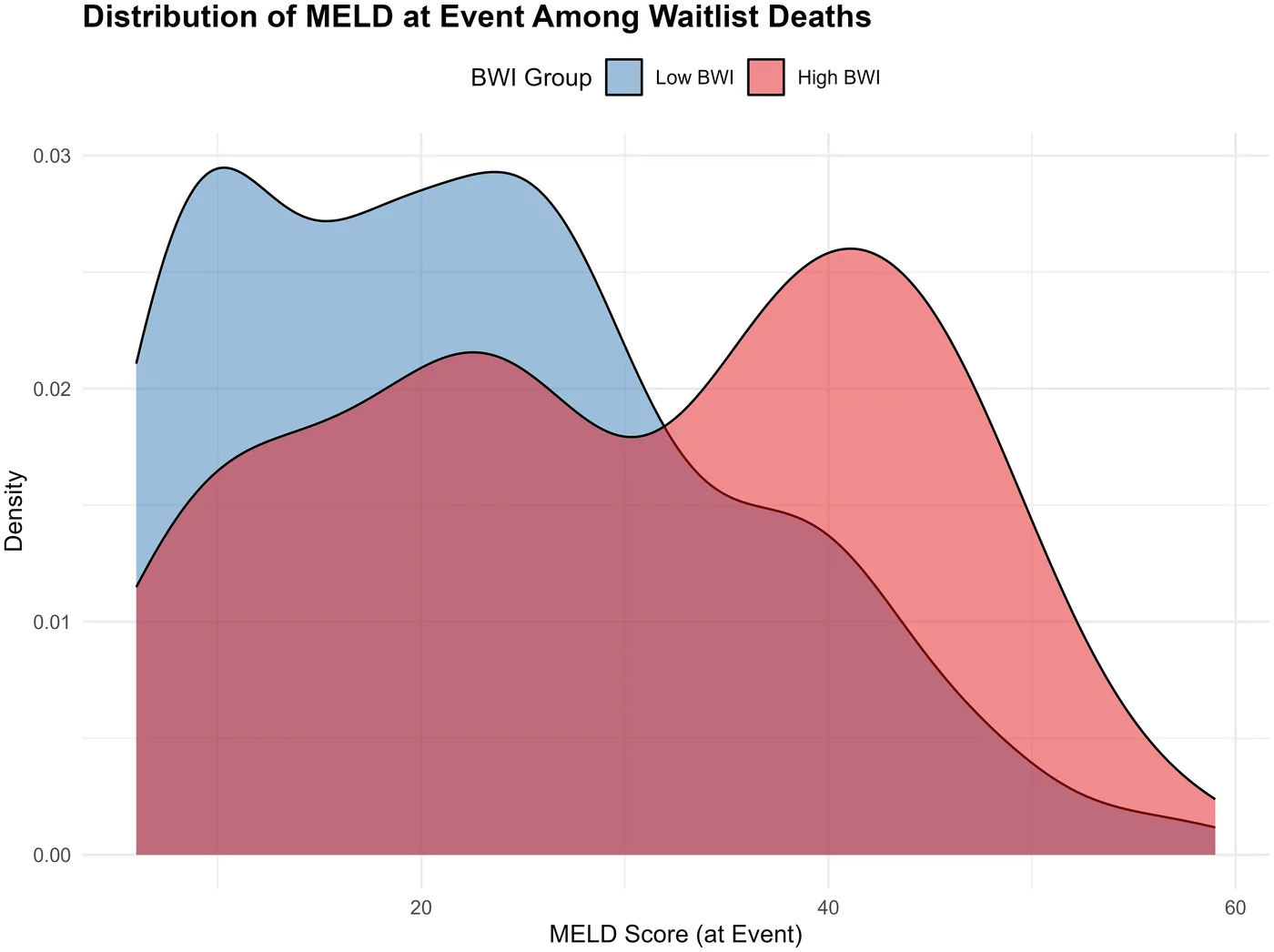
MELD at the time of death or clinical deterioration, stratified by BioWaitIndex. Kernel density plots showing the distribution of MELD scores at the time of death or removal because of clinical deterioration among candidates with below-median BWI (*n* = 256) and above-median BWI (*n* = 175), using the cohort median BWI of 2.85. Among the 431 candidates who experienced this outcome, 59.4% had below-median BWI. Candidates with below-median BWI experienced the outcome at lower MELD scores than those with above-median BWI (median 22 versus 30; *p* < 0.001) and remained on the waitlist longer (median 269 versus 121 days; *p* = 0.001). A graded pattern was also observed across BWI quartiles: median MELD at the time of the outcome increased from 19 in quartile 1 to 34 in quartile 4, whereas median waitlist duration decreased from 286 to 88 days (Kruskal–Wallis *p* < 0.001 and *p* = 0.003, respectively). These descriptive findings suggest that death or clinical deterioration may occur at comparatively lower MELD scores after longer waitlist exposure among candidates with lower BWI.

### Sex parity

To evaluate potential algorithmic bias, we performed sex-stratified competing risk models adjusted for race/ethnicity. BWI demonstrated consistent predictive performance across both groups. For transplantation, the hazard ratio was identical for males (HR 1.03, 95% CI 1.02-1.04, *p* < 0.001) and females (HR 1.03, 95% CI 1.02-1.04, *p* < 0.001). BWI was similarly associated with lower waitlist mortality for males (HR 0.98, 95% CI 0.97-0.99, *p* < 0.001) and females (HR 0.98, 95% CI 0.97-0.99, *p* < 0.001).

## Discussion

Our study introduces the BioWaitIndex (BWI), a machine learning-derived composite index of systemic physiologic reserve that enhances prediction of liver transplant waitlist outcomes. By distilling 12 routinely collected multisystem biomarkers into a single metric, BWI provides complementary prognostic information to the MELD score, particularly at the time of initial transplant evaluation when clinical decisions regarding listing strategy and physiologic optimization are most actionable.

While MELD remains the cornerstone of liver allocation, our findings highlight the importance of assessment timing of this acute severity measure. MELD was designed to predict 90-day mortality and relies on a narrow set of hepatorenal parameters (bilirubin, creatinine, INR, and sodium) (7). Our results demonstrate that MELD measured at initial evaluation was not significantly associated with subsequent death or clinical deterioration (sHR 1.01; *p* = 0.095), whereas BWI remained a robust independent predictor at the same timepoint (sHR 0.97, *p* < 0.001). This discrepancy reflects the fundamental difference in what each metric captures: MELD measures the acute severity of liver failure, BWI incorporates information about baseline multisystem physiologic status. The low correlation between the two scores (r = 0.26, r^2^ = 0.07) and their independent contributions to multivariable models suggest that they capture related but largely distinct dimensions of candidate status.

The distinct prognostic value of BWI was confirmed through reclassification analysis, which captures improvements in risk stratification often missed by traditional discrimination metrics. For the critical outcome of waitlist mortality, the addition of BWI to MELD, both measured at initial evaluation, yielded a net reclassification improvement (NRI) of 0.312 (*p* < 0.001). The continuous NRI reflects the net direction of changes in predicted risk rather than the percentage of candidates correctly assigned to discrete risk categories. A significant improvement was similarly observed for transplantation likelihood (NRI 0.111, *p* = 0.026), and results were similar when MELD assessed at the time of the outcome was used as the reference, supporting the conclusion that BWI contributes prognostic information beyond MELD within this cohort.

Feature importance analysis revealed that the leading contributors to BWI were from systemic rather than liver-specific markers. FEV1, systolic blood pressure, and blood urea nitrogen emerged as the leading contributors, spanning pulmonary, cardiovascular, and renal domains. In the transplant context, FEV1 likely captures respiratory muscle wasting, hepatopulmonary syndrome, and portopulmonary hypertension, all recognized contributors to waitlist mortality independent of MELD (34). Systolic blood pressure reflects hemodynamic reserve in the setting of cirrhotic cardiomyopathy and splanchnic vasodilation (35). BUN integrates renal function, nutritional status, and catabolic state, particularly relevant given the prevalence of hepatorenal syndrome (36).

BWI showed opposing associations with the two competing waitlist outcomes: higher BWI was associated with a greater likelihood of transplantation but a lower risk of death or clinical deterioration. This pattern should not be interpreted as higher BWI being protective. Rather, it may reflect current allocation dynamics. On a transplant waitlist, transplantation is an informative competing event influenced by clinical severity, allocation priority, organ availability, candidacy decisions, and center-level practice. Candidates whose clinical and biomarker profiles increase their probability of transplantation may undergo transplantation before they can experience waitlist death, thereby producing an inverse association with death or clinical deterioration. Conversely, candidates with lower BWI may appear less urgent by conventional measures, remain on the waitlist longer, and experience death or deterioration before their MELD score rises sufficiently to increase allocation priority. Because MELD functions as both a measure of short-term clinical acuity and an allocation tool, higher MELD is associated with both greater transplantation likelihood and greater risk of death or deterioration. BWI, which is measured at evaluation and does not influence allocation, may therefore provide complementary information about baseline vulnerability.

This interpretation was supported by the analysis of candidates who died or were removed because of clinical deterioration. Candidates with below-median BWI experienced the outcome at lower MELD scores than those with above-median BWI (median 22 vs. 30; *p* < 0.001; Figure 5) and remained on the waitlist for more than twice as long (median 269 vs. 121 days; *p* = 0.001). These findings suggest that BWI may help identify candidates whose waitlist vulnerability is not fully reflected by MELD at evaluation and who may benefit from closer surveillance or earlier reassessment.

Efforts to capture physiologic dimensions beyond organ-specific dysfunction in transplant recipients remain limited. Cremen et al. conducted a scoping review examining physiologic and immunologic biomarkers in solid organ transplant recipients and found that research is heavily biased toward kidney transplantation, which accounted for 23 of 32 included studies, while only three focused on liver transplant recipients (37). Among those limited liver studies, results regarding cellular markers such as telomere length were inconsistent (37). Molecular approaches including DNA methylation derived indices have shown preliminary feasibility in cirrhosis. LaHue et al. applied second-generation epigenetic indices (PhenoAge, GrimAge) in adults undergoing liver transplantation and observed trends toward greater physiologic compromise among frail subjects and those with poor post-transplant outcomes (38). However, this was a pilot study limited to 12 participants, did not reach statistical significance, and required specialized processing of peripheral blood mononuclear cells, limiting scalability (38). In contrast, BWI uses routinely collected clinical measurements and was evaluated in 1,502 waitlisted candidates without requiring specialized molecular assays.

Importantly, BWI is not a proxy for chronological age. Although the random forest model was trained to predict chronological age from biomarker patterns leveraging age as a training signal for systemic physiologic decline, the resulting index captures deviation from expected multisystem function. Two candidates of identical chronological age may have markedly different BWI values reflecting divergent systemic reserves. BWI is interpreted as deviation from expected biomarker patterns conditional on chronological age rather than as chronological age itself. Like other machine-learning age estimators, BWI may show a systematic inverse dependence on chronological age arising from regression to the mean (39); its associations with waitlist outcomes were retained after adjustment for chronological age (Supplementary Tables S9,10). BWI was also intentionally trained without liver-specific inputs so that it would capture multisystem physiologic status that is complementary to, rather than overlapping with, MELD. Future studies could examine whether incorporating liver-specific scores into the training target improves prediction.

These findings have several practical implications. First, BWI could serve as an early screening tool at the time of transplant evaluation to identify candidates who may benefit from closer monitoring or expedited reassessment despite moderate MELD scores. Second, the clear quartile stratification suggests BWI may help identify candidates for whom targeted nutritional, pulmonary, or kidney assessment could be considered, although the benefit of BWI-guided interventions requires prospective evaluation. Third, BWI's derivation from routine clinical data makes it potentially suitable for future electronic health record integration after multicenter validation in any transplant center with access to standard laboratory and spirometry data, without requiring machine-learning expertise from end users; at implementation, inputs would require unit harmonization and plausibility checks, and isolated missing values, including FEV1, could be handled with the same imputation approach used here. Fourth, BWI demonstrated similar associations across sexes, with identical hazard ratios for both males and females for transplantation and mortality, directly relevant to the ongoing policy debate around sex-based disparities in liver allocation (40). Future prospective studies should evaluate whether BWI-guided interventions at the time of evaluation improve post-transplant outcomes and whether BWI can be integrated into allocation algorithms as a complement to MELD.

This study has limitations. First, although BWI was independently clinically validated in a separate cohort of 1,502 liver transplant candidates, the clinical-validation cohort was retrospective and derived from a single transplant center. Additional prospective and multicentre external validation is therefore required before clinical implementation. Second, missing data remain an inherent challenge in real-world clinical datasets. Although imputed and observed distributions were comparable and the association between BWI and waitlist mortality was consistent in complete-case sensitivity analyses (Supplementary Tables S2,3), the missing-at-random assumption underlying multiple imputation cannot be verified. Third, NHANES data were analyzed as a simple random sample without survey weights or design variables, consistent with the BioAge framework from which the development data were drawn; because NHANES oversamples certain demographic groups, the learned biomarker–age relationships may not be nationally representative, and BWI could be systematically over- or under-estimated in subpopulations that were over- or under-represented in the development sample. Fourth, regional variations in transplant practices and organ availability may influence the specific hazard ratios reported. Finally, as with any machine learning-derived metric, ongoing validation across diverse populations and clinical settings is essential to establish robust clinical utility.

In conclusion, the BioWaitIndex represents a step toward more holistic risk stratification of liver transplant candidates. By leveraging routinely collected clinical data and machine learning, BWI provides prognostic information that complements the established MELD score, particularly at the time of initial evaluation when significant opportunity for clinical intervention exists. Integrating BWI at the time of evaluation could help identify candidates whose waitlist vulnerability is not fully captured by MELD alone.

## Data Availability

The original contributions presented in the study are included in the article/Supplementary Material, further inquiries can be directed to the corresponding author.
