# Supplementary material for: BioWaitIndex: a novel machine learning-derived biomarker for improved prediction of liver transplant waitlist outcomes
Source: Front Transplant. 2026 Oct 14;5:1964045. doi: 10.3389/frtra.2026.1964045 (PMC13713663; doi:10.3389/frtra.2026.1964045)
Supplement: Supplementary file 1 [file Datasheet1.docx]

Supplemental tables S1-S11

Supplemental Figure S1

TRIPOD AI Checklist

**Supplementary Table S1. Proportion of Participants With BWI Beyond ±2 SD of the Mean, Overall and by Age Decade, in the Development and Temporal-Validation Cohorts**

| Cohort / age group | n | Beyond ±2 SD, n (%) |
| --- | --- | --- |
| NHANES III (development) |  |  |
| Overall | 13,830 | 637 (4.6) |
| 20–29 | 3,073 | 116 (3.8) |
| 30–40 | 3,178 | 158 (5.0) |
| 41–50 | 2,094 | 92 (4.4) |
| 51–60 | 1,583 | 43 (2.7) |
| 61–70 | 1,833 | 70 (3.8) |
| 71+ | 2,069 | 95 (4.6) |
| NHANES IV (temporal validation) |  |  |
| Overall | 8,151 | 340 (4.2) |
| 20–29 | 1,487 | 60 (4.0) |
| 30–40 | 1,671 | 75 (4.5) |
| 41–50 | 1,551 | 71 (4.6) |
| 51–60 | 1,412 | 41 (2.9) |
| 61–70 | 1,262 | 45 (3.6) |
| 71+ | 768 | 32 (4.2) |

*BWI = random forest–predicted age minus chronological age. For NHANES III, predictions are out-of-bag (each participant predicted only by trees not trained on that participant); for NHANES IV, the fitted model was applied without retraining. Overall rows use the cohort mean ± 2 SD as cut-offs; age-group rows use the mean ± 2 SD within that group. Under a normal distribution, 4.6% of values fall beyond ±2 SD.*

**Supplementary Table S2. Missingness of Variables in the Institutional Cohort and Imputation Diagnostics**

| **Variable** | **Missing, %** | **Observed, mean (SD)** | **After imputation, mean (SD)** | **KS p** |
| --- | --- | --- | --- | --- |
| Albumin, g/dL | 2.8 | 3.01 (0.73) | 3.01 (0.72) | 1.00 |
| ALP, U/L | 2.8 | 167.9 (175.3) | 167.7 (173.9) | 1.00 |
| Creatinine, mg/dL | 2.6 | 1.65 (1.72) | 1.65 (1.72) | 1.00 |
| CRP, mg/dL | 26.0 | 2.79 (3.51) | 2.56 (3.45) | 0.03 |
| Glucose, mg/dL | 2.6 | 128.1 (56.7) | 127.8 (56.5) | 1.00 |
| Lymphocytes, % | 2.8 | 18.7 (10.3) | 18.7 (10.3) | 1.00 |
| MCV, fL | 2.7 | 95.6 (8.8) | 95.5 (8.9) | 1.00 |
| WBC, ×10³/µL | 2.7 | 7.34 (5.53) | 7.33 (5.50) | 1.00 |
| BUN, mg/dL | 2.7 | 25.7 (20.5) | 25.7 (20.5) | 1.00 |
| HbA1c, % | 7.5 | 5.35 (1.32) | 5.33 (1.32) | 1.00 |
| Systolic BP, mmHg | 3.3 | 125.9 (22.6) | 125.8 (22.7) | 1.00 |
| FEV1, L | 53.9 | 2.50 (0.83) | 2.41 (0.83) | 0.10 |

*Missingness is reported among the candidates evaluated for liver transplantation, in whom imputation was performed; waitlist analyses were restricted to those who were listed. Means and SDs are shown for observed values and for the completed dataset after multiple imputation; distributions were compared using the Kolmogorov–Smirnov (KS) test.*

**Supplementary Table S3. Sensitivity Analysis of BWI Associations by Missing-Data Handling**

| Analysis | n | Transplantation sHR (95% CI) | p | Death/Deterioration sHR (95% CI) | p |
| --- | --- | --- | --- | --- | --- |
| Full cohort, imputed | 1,259 | 1.02 (1.01–1.03) | <0.001 | 0.97 (0.97–0.98) | <0.001 |
| Complete cases (all 12 biomarkers observed) | 401 | 1.01 (1.00–1.02) | 0.15 | 0.98 (0.96–0.99) | 0.005 |
| FEV1 observed | 583 | 1.01 (1.00–1.02) | 0.14 | 0.98 (0.96–0.99) | <0.001 |
| FEV1 imputed | 676 | 1.02 (1.01–1.03) | <0.001 | 0.97 (0.96–0.98) | <0.001 |

Restricted to the 1,259 candidates with MELD available at initial evaluation, consistent with Table 2.

**Supplementary Table S4. Biomarker Profile Comparison: LT Evaluation Cohort vs. NHANES Training Cohort**

| **Characteristic** | **Evaluation (‘LT’),**  **N = 2,070** | **Training (‘NHANES’),**  **N = 13,830** | **p-value** |
| --- | --- | --- | --- |
| Age (years) | 60.1 (50.9, 66.3) | 43.0 (31.0, 63.0) | **<0.001** |
| Albumin (g/dL) | 3.0 (2.5, 3.5) | 4.1 (3.9, 4.4) | **<0.001** |
| Alkaline phosphatase (U/L) | 132.5 (94.0, 186.8) | 81.0 (67.0, 99.0) | **<0.001** |
| Blood urea nitrogen (mg/dL) | 18.0 (12.0, 32.0) | 13.0 (11.0, 17.0) | **<0.001** |
| C-reactive protein (mg/dL) | 1.3 (0.7, 3.1) | 0.2 (0.2, 0.4) | **<0.001** |
| Hemoglobin A1c (%) | 5.1 (4.5, 5.9) | 5.3 (5.0, 5.7) | **<0.001** |
| Systolic blood pressure (mm Hg) | 125.0 (112.0, 140.0) | 121.5 (111.0, 136.0) | **<0.001** |
| FEV₁ (L) | 2.4 (1.8, 2.9) | 2.9 (2.3, 3.6) | **<0.001** |
| Creatinine (mg/dL) | 1.0 (0.8, 1.7) | 0.8 (0.7, 1.0) | **<0.001** |
| Glucose (mmol) | 6.2 (5.2, 8.0) | 5.1 (4.7, 5.6) | **<0.001** |
| Lymphocytes (%) | 17.7 (10.7, 24.9) | 32.5 (27.1, 38.3) | **<0.001** |
| Mean corpuscular volume (fL) | 95.3 (89.9, 101.3) | 89.8 (86.6, 92.7) | **<0.001** |
| White blood cell count (×10³/µL) | 5.7 (4.1, 8.6) | 6.8 (5.7, 8.3) | **<0.001** |

Comparison of the 12 BWI biomarkers between the institutional liver transplant evaluation cohort and the NHANES training cohort. All values are reported as median (IQR). LT candidates demonstrated profoundly altered biomarker profiles characteristic of end-stage liver disease compared to the non-institutionalized participants (all p < 0.001). The institutional evaluation cohort (n = 2,070) is larger than the waitlisted cohort (n = 1,502) because not all candidates evaluated for liver transplantation proceed to waitlisting. BWI was calculated from biomarkers collected at the time of evaluation, but waitlist outcomes were analyzed only among the 1,502 candidates who were formally listed.

**Supplementary Table S5. Distribution of Labs Used in BWI Development by Waitlist Outcome**

| **Characteristic** | **Overall,**  **N = 1,502¹** | **Other/Censored,**  **n = 294¹** | **Death/Deteriorated,**  **n = 431¹** | **Transplant,**  **n = 777¹** | **p-value²** |
| --- | --- | --- | --- | --- | --- |
| Albumin, g/dL | 3.0 (2.5, 3.5) | 3.1 (2.7, 3.6) | 3.1 (2.6, 3.5) | 2.9 (2.5, 3.5) | 0.02 |
| ALP, U/L | 129.5 (94.0, 185.0) | 126.5 (92.0, 177.3) | 133.5 (95.0, 189.0) | 129.0 (93.3, 187.0) | 0.399 |
| Creatinine, mg/dL | 1.0 (0.8, 1.7) | 0.9 (0.7, 1.3) | 1.1 (0.8, 1.8) | 1.1 (0.8, 1.8) | **<0.001** |
| CRP, mg/dL | 1.5 (0.7, 3.5) | 1.1 (0.6, 2.6) | 1.5 (0.8, 3.3) | 1.7 (0.8, 3.9) | 0.001 |
| Glucose, mg/dL | 112.0 (93.0, 144.0) | 107.0 (92.0, 134.0) | 113.0 (92.0, 149.8) | 114.0 (95.0, 143.5) | 0.067 |
| Lymphocyte, % | 17.9 (10.9, 25.1) | 20.1 (14.8, 26.4) | 17.4 (10.4, 24.6) | 17.6 (10.2, 24.7) | **<0.001** |
| MCV, fL | 95.7 (90.3, 101.9) | 94.2 (89.6, 99.7) | 95.7 (90.3, 101.4) | 96.3 (90.7, 102.8) | 0.001 |
| WBC, ×10³/µL | 5.7 (4.0, 8.5) | 5.6 (4.1, 7.7) | 5.5 (4.0, 8.0) | 5.8 (4.1, 9.0) | 0.049 |
| BUN, mg/dL | 18.0 (12.0, 32.0) | 16.0 (11.0, 24.0) | 19.0 (13.0, 35.0) | 19.0 (13.0, 33.0) | **<0.001** |
| HbA1c, % | 5.1 (4.4, 5.8) | 5.1 (4.6, 5.7) | 5.2 (4.6, 6.0) | 5.0 (4.3, 5.8) | 0.007 |
| Systolic BP, mmHg | 125.0 (113.0, 140.0) | 128.0 (115.0, 147.0) | 122.0 (107.0, 136.5) | 126.0 (115.0, 139.0) | **<0.001** |
| FEV1, L | 2.5 (2.0, 3.1) | 2.6 (2.1, 3.1) | 2.3 (1.9, 3.0) | 2.5 (2.0, 3.1) | 0.012 |

All values are reported as median (IQR). ¹Median (IQR); n (%). ²Kruskal-Wallis rank sum test; Fisher’s Exact Test for Count Data.

**Supplementary Table S6. BWI Predictive Performance Stratified by MELD at Evaluation**

| MELD Stratum | Outcome | BWI HR (95% CI) | p-value |
| --- | --- | --- | --- |
| Low MELD (< 20) | Transplantation | 1.01 (1.00, 1.02) | **0.028** |
| Low MELD (< 20) | Death/Deterioration | 0.97 (0.96, 0.98) | **<0.001** |
| High MELD (≥ 20) | Transplantation | 1.02 (1.02, 1.03) | **<0.001** |
| High MELD (≥ 20) | Death/Deterioration | 0.98 (0.97, 0.99) | **<0.001** |

*Subdistribution hazard ratios for BWI from separate Fine-Gray competing risks models within each MELD stratum, adjusted for sex and race/ethnicity. BWI remained a significant independent predictor of waitlist mortality in both low- and high-MELD groups, confirming its prognostic value across the full spectrum of hepatic disease severity. In the low-MELD stratum, BWI was predictive of death/deterioration (HR 0.97, p < 0.001) and for transplantation (HR 1.01, p = 0.028), consistent with finding that the candidates with low allocation priority face underappreciated mortality risk identifiable by BWI.* All models adjusted for sex and race/ethnicity.

**Supplementary Table S7. Comparative Performance of Physiologic Indices for Waitlist Outcome Prediction**

| **Metric** | **Transplantation**  **HR (95% CI)** | **Transplantation**  **p-value** | **Death/Deterioration**  **HR (95% CI)** | **Death/Deterioration**  **p-value** |
| --- | --- | --- | --- | --- |
| **BioWaitIndex (BWI)** | 1.03 (1.02, 1.04) | **<0.001** | 0.98 (0.97, 0.98) | **<0.001** |
| MELD at Evaluation | 1.05 (1.04, 1.06) | **<0.001** | 1.00 (0.99, 1.01) | 0.56 |
| MELD at Event | 1.03 (1.03, 1.04) | **<0.001** | 1.02 (1.01, 1.03) | **<0.001** |
| Homeostatic Dysregulation | 1.10 (1.03, 1.16) | 0.002 | 1.08 (1.00, 1.17) | 0.052 |
| KDM Biological Age | 1.00 (1.00, 1.01) | <0.001 | 1.00 (1.00, 1.01) | 0.084 |
| Levine Phenotypic Age | 1.00 (0.99, 1.00) | 0.11 | 1.03 (1.02, 1.04) | **<0.001** |
| Body Mass Index (BMI) | 1.01 (1.00, 1.02) | 0.19 | 1.00 (0.98, 1.01) | 0.71 |

Subdistribution hazard ratios from separate Fine-Gray competing risks models for each metric, each adjusted for sex and race/ethnicity. BWI and MELD at Event were the only metrics to significantly predict both transplantation and death outcomes (p < 0.001 for both). BWI was the only metric demonstrating bidirectional predictive capacity, simultaneously predicting increased transplantation and decreased mortality, distinguishing it from all comparators. Established physiologic indices were included as benchmarks for comparative evaluation.

**Supplementary Table S8. Biomarker Composition and Exclusions in Biological Age Models**

| **Biomarker** | **PhenoAge** | **KDM** | **HD** | **BWI** |
| --- | --- | --- | --- | --- |
| Albumin | ✓ | ✓ | ✓ | ✓ |
| Alkaline Phosphatase (ALP) | ✓ | ✓ | ✓ | ✓ |
| Creatinine | ✓ | ✓ | ✓ | ✓ |
| C-Reactive Protein (CRP) | ✓ | ✓ | ✓ | ✓ |
| Glucose | ✓ | — | ✓ | ✓ |
| Lymphocyte Percent | ✓ | — | ✓ | ✓ |
| Mean Cell Volume (MCV) | ✓ | — | ✓ | ✓ |
| White Blood Cell Count (WBC) | ✓ | — | ✓ | ✓ |
| Blood Urea Nitrogen (BUN) | — | ✓ | ✓ | ✓ |
| HbA1c | — | ✓ | ✓ | ✓ |
| Systolic Blood Pressure | — | ✓ | ✓ | ✓ |
| FEV1 (Lung Function) | — | ✓ | ✓ | ✓ |
| Total Cholesterol | — | ✓ | — | — |
| Red Cell Dist. Width (RDW) | Excluded* | — | Excluded* | Excluded* |
| Cytomegalovirus (CMV) | — | Excluded* | Excluded* | Excluded* |
| Uric Acid | — | — | Excluded* | Excluded* |

*Excluded due to high missingness in the institutional cohort. Biomarker inclusion across physiologic index algorithms. ✓ indicates the biomarker is included in the algorithm; indicates the biomarker is not part of the standard formula for that algorithm.

**Supplementary Table S9. Age-Adjusted Fine–Gray Models With BWI and MELD Measured at Initial Transplant Evaluation**

| **Variable** | **Coefficient** | **SE** | **sHR** | **95% CI** | **p-value** |
| --- | --- | --- | --- | --- | --- |
| **Panel A: Outcome: Transplantation** |  |  |  |  |  |
| MELD at evaluation | 0.043 | 0.005 | 1.04 | 1.03, 1.05 | **<0.001** |
| BWI at evaluation | 0.013 | 0.004 | 1.01 | 1.00, 1.02 | **0.004** |
| Age at evaluation, per year | −0.008 | 0.004 | 0.99 | 0.98, 1.00 | 0.079 |
| Sex (Male) | 0.097 | 0.087 | 1.10 | 0.93, 1.31 | 0.27 |
| Race: Non-Hispanic Black | 0.044 | 0.161 | 1.05 | 0.76, 1.43 | 0.78 |
| Race: Hispanic | −0.055 | 0.104 | 0.95 | 0.77, 1.16 | 0.60 |
| Race: Non-Hispanic Asian | 0.119 | 0.188 | 1.13 | 0.78, 1.63 | 0.53 |
| Race: Other/Unknown | −0.418 | 0.424 | 0.66 | 0.29, 1.51 | 0.32 |
| **Panel B: Outcome: Death or Clinical Deterioration** |  |  |  |  |  |
| MELD at evaluation | 0.013 | 0.006 | 1.01 | 1.00, 1.03 | **0.040** |
| BWI at evaluation | −0.012 | 0.006 | 0.99 | 0.98, 1.00 | **0.028** |
| Age at evaluation, per year | 0.033 | 0.007 | 1.03 | 1.02, 1.05 | **<0.001** |
| Sex (Male) | −0.035 | 0.112 | 0.97 | 0.78, 1.20 | 0.75 |
| Race: Non-Hispanic Black | −0.073 | 0.228 | 0.93 | 0.59, 1.45 | 0.75 |
| Race: Hispanic | 0.095 | 0.132 | 1.10 | 0.85, 1.42 | 0.47 |
| Race: Non-Hispanic Asian | −0.292 | 0.290 | 0.75 | 0.42, 1.32 | 0.31 |
| Race: Other/Unknown | 0.440 | 0.362 | 1.55 | 0.76, 3.16 | 0.22 |

*Same specification as Table 2 with additional adjustment for chronological age at evaluation (n = 1,259). Abbreviations and reference categories as in Table 2.*

**Supplementary Table S10. Age-Adjusted Fine–Gray Models With BWI at Evaluation and MELD at the Time of Outcome**

| **Variable** | **Coefficient** | **SE** | **sHR** | **95% CI** | **p-value** |
| --- | --- | --- | --- | --- | --- |
| **Panel A: Outcome: Transplantation** |  |  |  |  |  |
| MELD at time of outcome | 0.028 | 0.003 | 1.03 | 1.02, 1.04 | **<0.001** |
| BWI at evaluation | 0.020 | 0.004 | 1.02 | 1.01, 1.03 | **<0.001** |
| Age at evaluation, per year | −0.007 | 0.004 | 0.99 | 0.99, 1.00 | 0.080 |
| Sex (Male) | 0.157 | 0.081 | 1.17 | 1.00, 1.37 | 0.051 |
| Race: Non-Hispanic Black | 0.116 | 0.141 | 1.12 | 0.85, 1.48 | 0.41 |
| Race: Hispanic | −0.097 | 0.094 | 0.91 | 0.75, 1.09 | 0.30 |
| Race: Non-Hispanic Asian | 0.122 | 0.167 | 1.13 | 0.81, 1.57 | 0.46 |
| Race: Other/Unknown | −0.277 | 0.406 | 0.76 | 0.34, 1.68 | 0.50 |
| **Panel B: Outcome: Death or Clinical Deterioration** |  |  |  |  |  |
| MELD at time of outcome | 0.025 | 0.005 | 1.03 | 1.02, 1.03 | **<0.001** |
| BWI at evaluation | −0.015 | 0.005 | 0.98 | 0.98, 0.99 | **0.002** |
| Age at evaluation, per year | 0.029 | 0.006 | 1.03 | 1.02, 1.04 | **<0.001** |
| Sex (Male) | 0.013 | 0.101 | 1.01 | 0.83, 1.23 | 0.90 |
| Race: Non-Hispanic Black | −0.026 | 0.198 | 0.97 | 0.66, 1.44 | 0.90 |
| Race: Hispanic | 0.121 | 0.116 | 1.13 | 0.90, 1.42 | 0.30 |
| Race: Non-Hispanic Asian | −0.356 | 0.265 | 0.70 | 0.42, 1.18 | 0.18 |
| Race: Other/Unknown | 0.399 | 0.377 | 1.49 | 0.71, 3.12 | 0.29 |

*Same specification as Table 3 with additional adjustment for chronological age at evaluation (n = 1,480). Abbreviations and reference categories as in Table 3.*

**Supplementary Table S11. Cause-Specific Hazard Models for BWI, Complementing the Fine–Gray Analyses**

| **Model** | **Transplantation HR (95% CI)** | **p** | **Death/Deterioration HR (95% CI)** | **p** |
| --- | --- | --- | --- | --- |
| BWI + MELD at evaluation | 1.01 (1.00–1.02) | 0.001 | 0.98 (0.97–0.99) | <0.001 |
| BWI + MELD at time of outcome | 1.03 (1.02–1.03) | <0.001 | 0.99 (0.98–0.99) | 0.001 |

Cause-specific hazard ratios from Cox proportional-hazards models in which the competing waitlist outcome was treated as censoring, adjusted for sex and race/ethnicity. CI, confidence interval; HR, cause-specific hazard ratio; BWI, BioWaitIndex; MELD, Model for End-Stage Liver Disease.

Supplementary Figure S1. Calibration plots for BWI-based Fine–Gray competing-risk models predicting (a) liver transplantation and (b) waitlist death or clinical deterioration at one year. Predicted risks were grouped into deciles; observed risks were estimated using the Aalen–Johansen cumulative incidence function.
